# Supplementary material for: Mycobacterium tuberculosis Small RNA MTS1338 Confers Pathogenic Properties to Non-Pathogenic Mycobacterium smegmatis
Source: Microorganisms. 2021 Feb 17;9(2):414. doi: 10.3390/microorganisms9020414 (PMC7921967; doi:10.3390/microorganisms9020414)
Supplement: Supplementary file 1 [file microorganisms-09-00414-s001.zip › microorganisms-1115247 sub-supp/Supplementary Figure S1.pptx]

## Slide 1
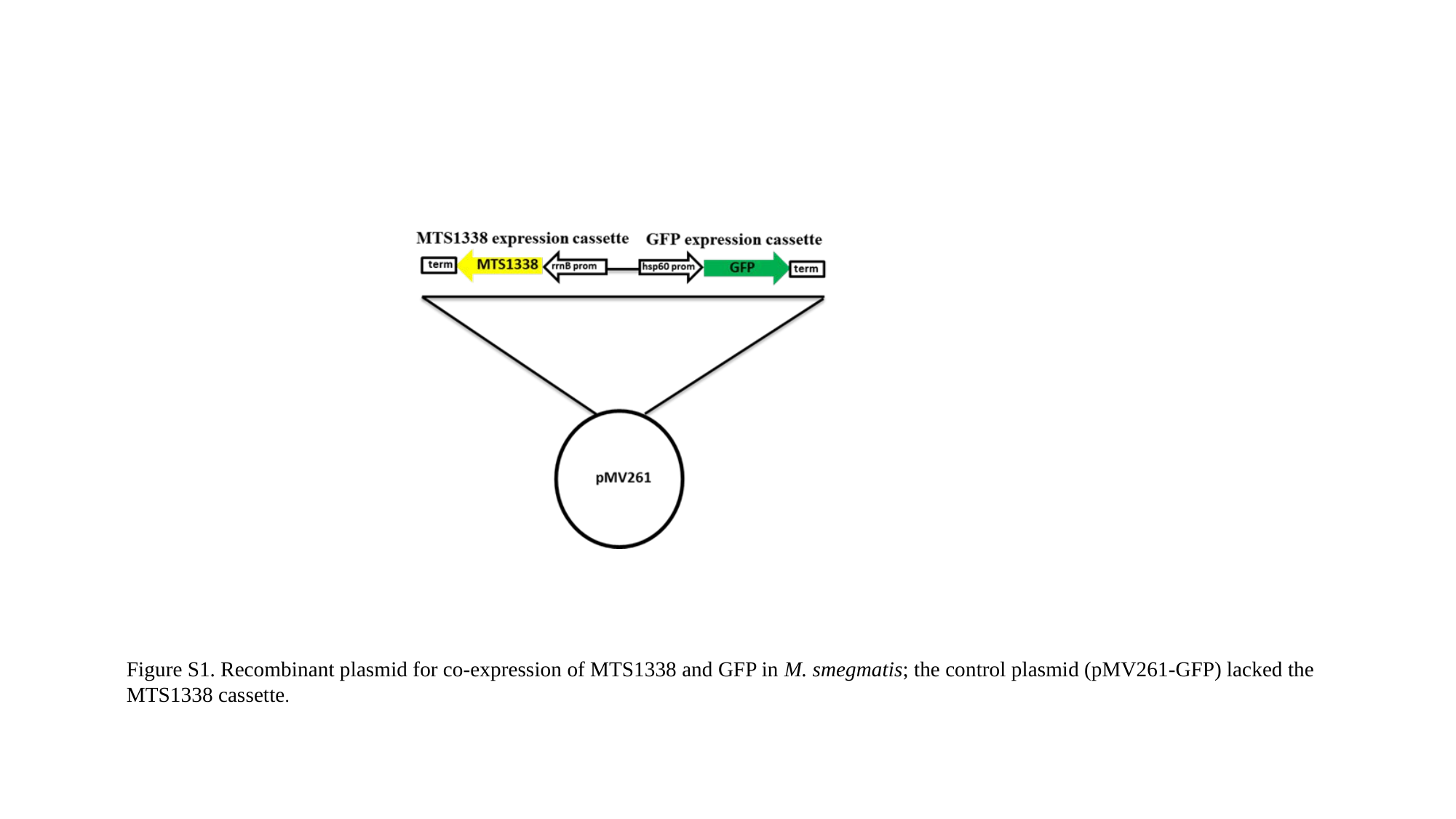

Figure S1. Recombinant plasmid for co-expression of MTS1338 and GFP in M. smegmatis; the control plasmid (pMV261-GFP) lacked the MTS1338 cassette.
