# Supplementary material for: Mycobacterium tuberculosis Small RNA MTS1338 Confers Pathogenic Properties to Non-Pathogenic Mycobacterium smegmatis
Source: Microorganisms. 2021 Feb 17;9(2):414. doi: 10.3390/microorganisms9020414 (PMC7921967; doi:10.3390/microorganisms9020414)
Supplement: Supplementary file 1 [file microorganisms-09-00414-s001.zip › microorganisms-1115247 sub-supp/Supplementary Figure S2.pptx]

## Slide 1
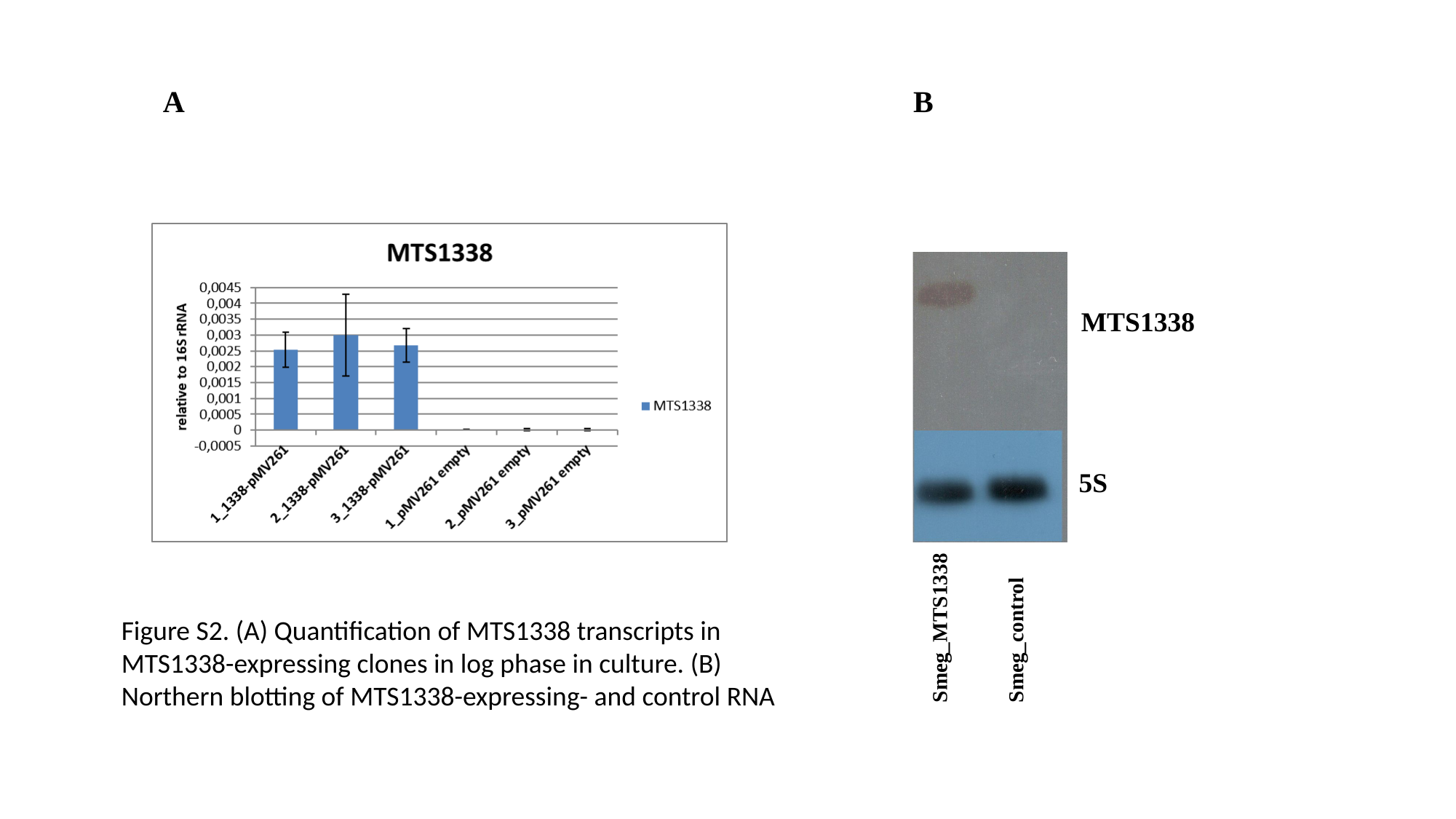

A
B
MTS1338
5S
Smeg_MTS1338
Smeg_control
Figure S2. (A) Quantification of MTS1338 transcripts in MTS1338-expressing clones in log phase in culture. (B) Northern blotting of MTS1338-expressing- and control RNA
