# Supplementary material for: Mycobacterium tuberculosis Small RNA MTS1338 Confers Pathogenic Properties to Non-Pathogenic Mycobacterium smegmatis
Source: Microorganisms. 2021 Feb 17;9(2):414. doi: 10.3390/microorganisms9020414 (PMC7921967; doi:10.3390/microorganisms9020414)
Supplement: Supplementary file 1 [file microorganisms-09-00414-s001.zip › microorganisms-1115247 sub-supp/Supplementary Table S1.docx]

**Supplementary Table 1. Oligonucleotides used in the study**

| Name | Sequence, 5’-3’ |
| --- | --- |
| MTS1338-for | GTGCTGGGCGATTGAGC |
| MTS1338-rev | GCGGTAGCCCCGTCTT |
| 16S-for | ATGTCGGTTCCCTTGTGGC |
| 16S-rev | CAAGGGTTGCGCTCGTTG |
| MSmeg-0965_for | aaccgtcttacccgtgagtg |
| MSmeg-0965_rev | ggggtggtgtagctgaagtt |
| MSmeg-5872_for | actacgtgaccaagccgttc |
| MSmeg-5872_rev | atcttgggcttggacaacac |
| MSmeg-3886_for | ggagtcttcggcttcaacag |
| MSmeg-3886_rev | cacagctggtacagccacac |
| MSmeg-2433_for | gctggtcaaccagaacgagt |
| MSmeg-2433_rev | tctttcaccaggccgtacat |
| MSmeg-0615_for | attcctcgacaccaacgaag |
| MSmeg-0615_rev | gtgaacagacgctccatgtc |
| MSmeg-5244_for | acatcaagggcatggaactc |
| MSmeg-5244_rev | gcgatctgtttgttggtcag |
| MSmeg-3935_for | aggatcattcgtcgcaagtc |
| MSmeg-3935_rev | ggtttctcgaacggactcag |
| mIL1-β-for | CAACCAACAAGTGATATTCTCCATG |
| mIL1-β-rev | ATCCACACTCTCCAGCTGCA |
| mTGF-β-for | accgcaacaacgccatcta |
| mTGF-β-rev | gcgtatcagtgggggtcag |
| mIL6-for | accagaggaaattttcaataggc |
| mIL6-rev | tgatgcacttgcagaaaaca |
| mIL10-for | tgtcaaattcattcatggcct |
| mIL10-rev | atcgatttctcccctgtgaa |
| mTNF-α-for | ctgaacttcggggtgatcg |
| mTNF-α-rev | ggcttgtcactcgaattttgaga |
| mIL12-for | tgtcaatcacgctacctcctc |
| mIL12-rev | tcgggactggctaagacac |
| mIL4-for | gttgtcatcctgctcttctttctc |
| mIL4rev | cactctctgtggtgttcttcgt |
| m_actin-β-for | gatcaagatcattgctcctcctg |
| m_actin-β-rev | acgcagctcagtaacagtcc |
